# Progress in Medicine

**Published:** 1895-08-17

**Authors:** 


### Progress in Medicine.

### RHEUMATISM AND GOUT.

**Acute Rheumatism.**—The active cause of acute rheumatism has long been the subject of various theories, none of which have been particularly well supported by facts. However, a brilliant attempt has now been made to show that "it is a specific febrile disease caused by the introduction from without of a pathogenic microbe, and its multiplication in the system," one which is endemic in most parts of the world, but which at times becomes epidemic or pandemic. The Milroy lecturer, Arthur Newsholme, collected an immense amount of evidence from the chief hospitals of the world and from the notification returns in Scandinavia, showing that actual epidemics do occur frequently, and in some years almost universally. These outbreaks appear to come when the rainfall is deficient, but only then if the ground water is particularly low and the temperature of the soil high. In fact, the view these well-supported arguments lead to is that the active microbe is one of saprophytic nature which develops special virulence when "incubated" in a warm, dry soil. The analogy of the symptoms to those of known specific fevers is shown by the mode of onset, often with wandering pains and sore throat; endocarditis, hyperpyrexia, and by the action of salicylates, which are powerful antiseptics. The ill-defined duration of the fever is largely due to secondary serous inflammations, the tendency to relapses and second attacks is parallel to what is seen in erysipelas, and the importance in it of personal predisposition is seen also in certain other fevers. Again, while some fevers are only infectious through the excreta, in rheumatic fever the poison being buried in the joints is only communicable with difficulty. Evidence has also been accumulated that the poison clings to certain houses, and in epidemics the disease gradually spreads from certain centres over a given tract of country, but whether there is infection from case to case, or whether like malaria it starts each time from the soil is unknown.

The lectures, indeed, are full of an enormous number of facts which it is most difficult to summarise. One more topic may be selected, viz., the direct bacteriology of the disease. A number of organisms have been found in the synovial fluids and pericardial exudations, sometimes of unknown kinds, sometimes staphylo and strepto cocci, but as Dr. Newsholme says, the evidence in favour of any one of them being the materies morbi is unsatisfactory.[1] Sacaze, indeed, records a case of rheumatic fever which came on a week after a wound of the foot in which staphylococci albi were found, and believes that such septic wounds are often the precursors of attacks; among the commonest are tonsillar inflammations.[2] Leyden, too, has isolated certain diplococci from endocarditis in cases of rheumatism which grow only on human blood serum.[3] Yet no instance of the communication of the disease by inoculation has yet been found of indisputable nature, though E. D. Williams reports a case where acute rheumatism with chorea, endocarditis, and transient pneumonia followed tattooing and commenced in the arm operated on.

A. Robin and Leredde[4] describe also a typhoid type of acute rheumatism in which all the symptoms of typhoid may occur, except the pink spots and tympanites. That it is a true rheumatism is shown not only by the effect of salicylates and the presence of joint affections, but by the urine containing a normal amount of phosphoric acid, its pigmented sediment, the ucrobilinuric red colour and the frequent absence of albumen.[5] Suppurative arthritis may appear in acute rheumatism, according to Mauclaire, though it is probable that the suppuration is due to a secondary infection. Other instances of joint suppuration may be either of a pseudo-rheumatic character where the joint merely shows the first local sign of a general infection, or gonorrhœal in which the pathology is, indeed, uncertain.[6]

**Rheumatoid Arthritis.**—At least three views have been put forward as to the nature of this disease. It has been regarded as a joint lesion dependent on a phthisical inheritance, or one arising in gouty families, or a nerve lesion which secondarily causes the arthritis and muscular wasting. The last theory is supported by Spender, of Bath, who would regard it as a form of progressive muscular atrophy, marked in early stages by rapid pulse, pigmentation of the skin, and pain in the ball of the thumb. Folli has reported four cases, in three of which marked changes were found in the anterior cornua, consisting of atrophy of the cells, diminution of their number, and rarefaction of the neuroglia. These lesions were so advanced that he thinks them primary and not due to the changes in the limbs.[7] Jonathan Hutchinson looks upon an inherited tendency to gout as the root of the disease, but there may be a legacy of scrofula also, so that it is often difficult to say how much belongs to one and the other disease. Joint swellings in young people of the former type may reach an early stage, and then pass off entirely, so that the decision between a strumous and an arthritic swelling is important not only for treatment, but also for prognosis.

**Treatment.**—Pagan Lowe, distinguishing senile rheumatic arthritis[8] from rheumatoid, has noticed that in the former the joint is flexed almost from the first, and recommends forcible extension to break down adhesions. In mild cases the patient can walk at once without pain after this treatment. Persevering exercises afterwards are desirable in all cases. We recently noticed Latham's rules for the successful administration of salicylates. H. Huchard[9] has also laid down that it should be given in large quantities from the beginning of the attack, but divided into small frequent doses, and that it should be continued after the pains have ceased for twelve days or more. Thus he gives at least 90 grains daily for the first two days, especially since he believes it can prevent cardiac complications, though it does not cure them when existing. Frequent small doses are needed on account of the rapid elimination of the drug, and the quick changes and developments of the disease. If albuminuria exists before the attack salicylates are counter-indicated, but not if it merely comes on during the fever. In all cases the remedy should be well diluted, and when in a serious type of rheumatism the pains disappear but the fever remains high the drug must not be left off.[10] Solis Cohen recommends in chronic cases and in acute tonsilitis a salicylate of iron mixture. Salicylate of soda (℥ss.) is dissolved in a solution of citrate of ammonia (℥jss.), to which an excess of citric acid has been added. Tincture of iron (℥ss.) can now be mixed with it without producing the usual black precipitate.[11] This is of great value in anæmic patients. H. C. Wood has used strontium salicylate with success in sub-acute cases and tested its effects on the heart. The dose required to affect the blood pressure is twice that of the soda salt, so that it is much safer; besides, it improves instead of injuring the digestion, and is a most efficient intestinal antiseptic.[12] Salophen in doses of 30 grains daily was found by H. Lavrand to be efficacious without causing indigestion or cinchonism. Most, however, of his cases were chronic.[13] Marié gives 60 grains divided into six doses. Much larger doses are said to be useless as they pass unchanged through the body. He found it efficacious in acute rheumatism, gout, chorea, and other diseases, and especially valuable in a case of rheumatism with severe cardiac complications, where salicylates aggravated the heart symptoms. The absence of any digestive disturbance after its use and the tastelessness of the drug are strong points in its favour.[14] Lactophenin is another remedy which claims to produce the good effects of the salicylates without their disadvantages. It is given in doses of eight grains to the extent of 60 or more grains in the day as an antipyretic and in 15 grain doses as a hypnotic. Von Roth found that under its use the pains and swelling disappeared in one or two days, and the temperature was permanently depressed. Some observers have noted occasional cyanosis under its use, though without any definite depressing effect on the heart or respiration. Diuresis remains normal, and the pulse becomes fuller and slower.[15] Von Roth treated 28 cases with it, and in his paper summarises the reports of previous authorities.

**Gout.**—The pathology has been placed in an entirely new light by the investigations of Horbaczewsky and others on the formation of uric acid. Uric acid has been regarded as a product of proteid digestion and metabolism, and, as a relic of a primitive condition in which it took the place of urea entirely. Haig, too, endeavoured to show that in health the result of this metabolism was the excretion of urea and uric acid in a fixed proportion of about 33 to 1. Horbaczewsky and his pupils find, however, that the excretion of urea fluctuates with the food digested, while uric acid is comparatively constant in each individual. It is increased, however, by anything producing leucocytosis and destruction of the white corpuscles, as in pneumonia, leucocythœmia, extensive burns, and by the use of alcohol, pilocarpine, or by excessive exercise, while urea is unaltered. It is, in short, a product of the nuclein of the white corpuscles. This appears to be confirmed by the phenomena of digestion, which is usually accompanied by a temporary leucocytosis, for a rise of uric acid excretion follows a meal in two to five hours, while the increase of urea takes place only after metabolism has occurred, that is some time later. Horbaczewsky has formed uric acid by breaking up the nuclein of the spleen pulp, and, indeed, it can be prepared from the nuclein of any and every tissue in the body. In children, again, where the proportion of white to red blood corpuscles is greater than in adult life, we find a similar increase in the uric acid excreted. The source of the acid is certainly not the kidneys alone, but whether it is principally derived from the liver, or from some other organ where the white corpuscles are chiefly broken down, is not certain.

Minkowsky appeared to have shown that it arises from the liver, but other investigations cast some doubt upon this. Levison has summarised the recent work on the production of uric acid in his volume on "The Uric Acid Diathesis," which is discussed by Vaughan Harly[16] and others.[17] The examination of the uric acid in the blood by Von Jaksch some time ago confirmed these later results, for he not only found an increase in diseases where leucocytosis was present, but also in kidney disease where elimination was imperfect. It is to a previous kidney failure that the new school attribute the uric acid phenomena of gout together with perhaps some special cause for an excessive destruction of white corpuscles. The question whether the uric acid excretion is increased when it is given in food is variously debated, and Haig maintains that the different results obtained depend on the neglect of the laws which govern its solubility in the blood. Thus, where the acidity of the blood is great the uric acid is quickly swept out of the circulation, and where it is injected into the blood direct, and the animal bled to death within a few minutes, there is hardly any to be found in the fluid. The excretion depends on the amount which can be kept in circulation, and this is greater when an excess of alkalies or salicylates are present.[18]

[1] Lanc., March 9, 1895. [2] Am. J. Med. Sc., Feb. [3] Deutsch. Med. Woch, Dec. 6, 1894. [4] B. M. J., June 29, 1895. [5] Med. Week, Sep. 1, 1894. [6] B. M. J., March 16, 1895. [7] B. M. J., Feb. 2, 1895. [8] Clin. Jour., May 29. [9] B. M. J., Nov. 3, 1894. [10] N. Y. Med. Jour., Jan. 12, 1895. [11] Ther. Gazette, Jan. [12] N. Y. Med. J., Jan. 12. [13] B. M. J., April 6. [14] Med. Week., June 8. [15] Ther. Gazette, Jan. [16] B. M. J., March 23. [17] Med. Rec., April 6. [18] B. M. J., Dec. 18, 1894.

*(To be continued.)*

